# Concentration, Distribution and Biomagnification of Novel Brominated Flame Retardant in Grassland Food Chain and Sheep from Inner Mongolia, China

**DOI:** 10.3390/ijerph191912785

**Published:** 2022-10-06

**Authors:** Wenming Chen, Te Bu, Tianwei Li, Junsong Bao, Ying Wang, Jicheng Hu, Jun Jin

**Affiliations:** 1 College of Life and Environmental Sciences, Minzu University of China, Beijing 100081, China; 2 Beijing Food and Environmental Health Engineering Center, Beijing 100081, China

**Keywords:** novel brominated flame retardants, biomagnification, grassland food chain, organ distribution

## Abstract

Novel brominated flame retardants (NBFRs) have been of great concern in the past few years due to their ubiquity in the environment and potential bioconcentration characteristics. This study takes Xilingol grassland in Inner Mongolia as the research area to analyze the pollution characteristics of NBFRs (pTBX, HBB, PBT, PBBz, and PBEB) in the grassland food chain. pTBX was more likely to be biomagnified in the food chain of amphibians, reptiles, and birds, whereas PBT and HBB were more likely to be biomagnified in the food chain of mammals. This may be because these animals have different diets and metabolic patterns. According to the concentration distribution of NBFRs in sheep organs and tissues, PBT, HBB, and PBBz easy bioaccumulated in sheep. The biomagnification effect of sheep adipose tissue and internal organs on NBFRs was inconsistent, so the biomagnification of chemicals in organisms cannot be determined only by the biomagnification of adipose tissue.

## 1. Introduction

Brominated flame retardants are widely used in electronic products, furniture, and polymers [1]. Some commonly used brominated flame retardants, such as polybrominated diphenyl ethers, persist in the environment and are toxic to animals, including humans [2,3]. Commercial pentabromodiphenyl ether, octabromodiphenyl ether, and decabromodiphenyl ether are classed as persistent organic pollutants, so the Stockholm Convention has banned the production and use of these products [4]. Novel brominated flame retardants (NBFRs) are being produced and used as replacements for polybrominated diphenyl ethers [5]. NBFRs such as hexabromobenzene (HBB), pentabromobenzene (PBBz), pentabromoethylbenzene (PBEB), pentabromotoluene (PBT), and tetrabromo-*p*-xylene (pTBX) are used industrially as additive flame retardants [6]. These NBFRs do not form chemical bonds with the products they are added to, so they are released into the environment more readily than reactive brominated flame retardants [7]. NBFRs have been detected in sewage sludge, sediment, indoor dust, aquatic biota, wild animal tissues, and human serum [8,9,10,11,12,13]. Some NBFRs have been found to be endocrine disruptors [14]. Therefore, attention is being paid to determining whether currently used NBFRs have the characteristics of persistent organic pollutants.

Bioaccumulation means that a chemical is absorbed by an organism from environmental media and food and reaches higher concentrations in the tissues of the organism than in the environmental media or food. Persistent organic pollutants (particularly polybrominated diphenyl ethers) have different bioaccumulation capacities in aquatic and terrestrial systems [15]. Animal models such as sheep are a good choice to study the bioaccumulation of organic pollutants. However, few studies on NBFRs biomagnification in terrestrial food chains have been performed. There are few studies on the distribution of chemical compounds in the animal organs and tissues used to determine their bioaccumulation. The Inner Mongolia grassland is far from industrial areas and is sparsely populated, so it was used as the study area in the research described here. The NBFRs concentrations in environmental media (air, soil, and water) and biota samples (grass, insect, lizard, snake, toad, bird, mouse, weasel, cattle, horse, sheep, and human) from the study area were determined to assess the biomagnification of the NBFRs by calculating biomagnification factors for the food chain. NBFRs bioaccumulation and biomagnification in the biota of the grassland ecosystem were studied systematically. The aim was to determine which NBFRs are biomagnified through the food chain. Studying the distribution of NBFRs in sheep tissues can further improve our understanding of the bioaccumulation of NBFRs in organisms.

## 2. Materials and Methods

### 2.1. Sampling

**Figure 1:**
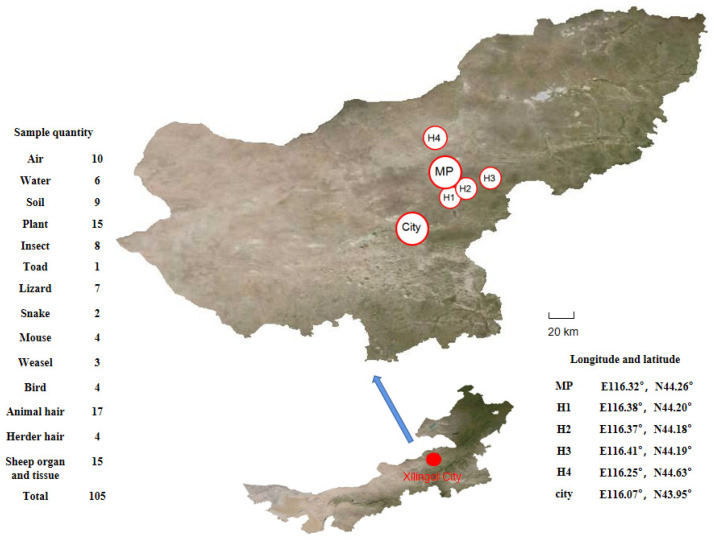
Map showing the sampling sites on the Xilingol grassland (Inner Mongolia, China).

Samples were collected from Xilingol grassland, Inner Mongolia, China. The sampling area was 40 km northeast of Xilingol City and included Maodeng pasture and four private pastures (labeled H1, H2, H3, and H4). The sampling points are shown in Figure 1.

A total of 105 samples were collected in mid-July 2018. The abiotic samples were groundwater, surface soil (0–10 cm deep), and ambient air (collected using a passive air sampler deployed for 80 d with an expected particulate phase collection rate of 3.5 m^3^/d [16]). The plant samples were *Allium mongolicum*, *Artemisia frigida*, *Eleusine indica*, and *Leymus chinensis*. The insect samples were carabidae, locust, moth, and scarab. The herbivore samples were rat and sparrow. The carnivore samples included cuckoo, lizard, swallow, toad, weasel, and snake. Most of the bird, snake, and weasel samples were collected from carcasses found on the roadside. The insect, lizard, mouse, and toad samples were captured. The feeding habits of the animals, including lizards [17], birds [18,19], toads [20], mice [21,22], weasels [23], and snakes [24] were determined by examining previous publications. The predation relationships were determined by assessing the contents of the predators’ stomachs. Cattle hair, horse hair, human hair, and wool samples were also collected. Two 1-year-old sheep (1-year-old sheep were sold by herdsmen for mutton. Food source of sheep on the grassland is herbage.) were collected from remote Xilingol grassland, Inner Mongolia, China (H4). We collected the samples of brain, heart, kidney, liver, spleen, lung, small intestine, stomach, chest muscle, hind leg muscle, abdominal fat, and tail fat from a whole sheep. Samples of tail fat and hind leg muscle from a sheep of the same breed raised by another herdsman were also collected. The samples were stored in the laboratory at −20 °C until they were prepared for analysis.

### 2.2. Chemical Analysis

The chemical reagents used and the sample preparation processes were described in detail in a previous publication [25,26]. Briefly, 2.5 ng of a ^13^C-labeled BDE-139 and 2.5 ng of ^13^C-labeled HBB were added to a water sample to act as internal standards, then the sample was liquid–liquid extracted. The extract was evaporated to 5 mL. Each solid sample (soil, plant and animal) was freeze-dried, ground, and then Soxhlet extracted. Then, 3 g aliquot of a ground sample was spiked with the internal standards (2.5 ng of ^13^C-labeled BDE-139 and 2.5 ng of ^13^C-labeled HBB) and Soxhlet extracted with 200 mL mixed solution of n-hexane and acetone (1:1, *v*:*v*) for 24 h. Each extract was passed through a composite silica gel column (Fill 1.0 g neutral silica gel, 4.0 g basic silica gel (NaOH 30% *w*/*w*), 2.0 g neutral silica gel, 8.0 g acidic silica gel (H_2_SO_4_ 44% *w*/*w*), 2.0 g neutral silica gel, 4.0 g anhydrous sodium sulfate from bottom to top) and then evaporated and transferred into 100 μL of nonane before instrumental analysis.

The sample extracts were analyzed using an Agilent 6890 gas chromatograph and an Agilent 5975N mass spectrometer (Agilent Technologies, Santa Clara, CA, USA). Separation was achieved using a DB-5-MS column (15 m long, 0.25 mm i.d., 0.1 μm film thickness; Agilent Technologies). Splitless injection mode was used, and the injection volume was 1 μL. The gas chromatograph oven temperature program started at 100 °C and increased to 300 °C, which was maintained for 22 min. Mass spectrum conditions: Negative chemical ionization source (NCI), selected ion mode (SIM) for quantitative analysis. The detected ions (*m*/*z*) of PBBz, PBT, PBEB, HBB, and PBBA were 471.6/473.6, 485.6/487.6, 499.6/501.6, 547.5/549.5, and 485.6/487.6, respectively. The detected ions (*m*/*z*) of ^13^C-labeled HBB and ^13^C-labeled BDE-139 were 559.5/561.5 and 573.6/575.6, respectively.

### 2.3. Quality Control and Assurance

Quantification was achieved using an internal standard method. The correlation coefficients for the standard curves for all of the analytes were >0.9995. The analyte concentrations in all of the sampling, transportation, solvent, and procedural blank samples were below the instrument detection limits. Limits of quantification (LOQs) were calculated with the signal-to-noise of 10, which the LOQs of NBFRs in air, water, soil, and biological samples were 0.01–0.14 pg/m^3^, 0.01–0.27 pg/L, 0.21–4.14 pg/g dw and 0.55–11.01 pg/g dw, respectively. The recoveries (mean ± standard deviation) for the abiotic samples, plant samples, and animal samples were 99.7% ± 31.1%, 59.4% ± 15.2%, and 60.1% ± 24.9%, respectively. Parallel samples were set up to evaluate experimental errors. For grassland food chain samples, the relative standard deviation (RSD) for each pair of parallel samples was <10%. For sheep tissue samples, the RSD values for the target analyte concentrations found in two liver samples and two hind leg muscle samples from the same sheep were 0.126% and 9.96%, respectively. The RSD values for the target analyte concentrations found in three hind leg muscle samples and two tail fat samples from different sheep were 7.04% and 2.86%, respectively.

All data analyses were performed using Graphpad prism 5 and Origin 2018 software. The data were rounded to three significant figures. Before a statistical test, each concentration below the limit of quantitation was replaced with half the limit of quantitation.

## 3. Results and Discussion

### 3.1. NBFRs Concentrations in the Xilingol Grassland Samples

**Figure 2:**
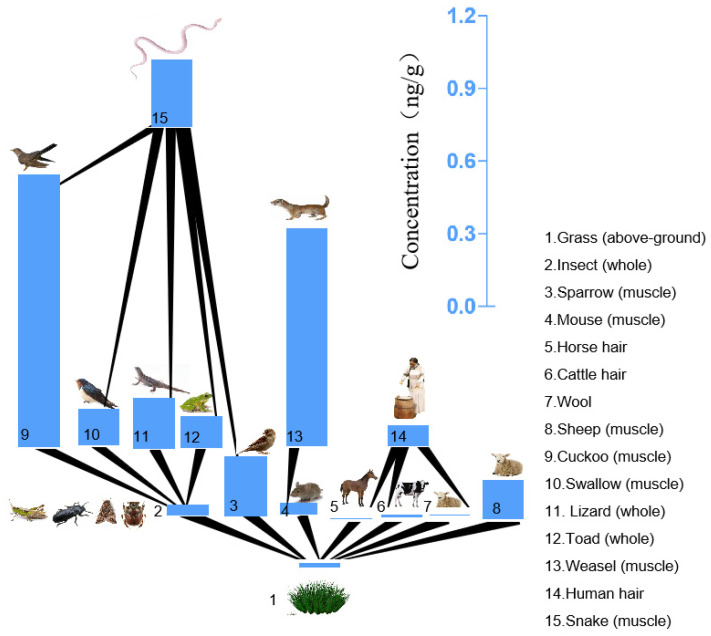
Novel brominated flame retardant concentrations found in the samples from the Xilingol grassland, China. Notes: The concentrations in (1) grass, (5) horse hair, (6) cattle hair, (7) wool, and (14) human hair are in ng/g dry weight and the concentrations in (2) insects, (3) sparrow, (4) mouse, (8) sheep, (9) cuckoo, (10) swallow, (11) lizard, (12) toad, (13) weasel, and (15) snake are in ng/g lipid weight.

The NBFRs were detected in the environmental media and biota samples from the Xilingol grassland. The detection rates of PBBz, PBT, pTBX, PBEB, and HBB were 96.7%, 96.7%, 82.4%, 78.0%, and 69.2%, respectively. This indicates that Xilingol grassland was generally polluted by NBFRs. The mean total NBFRs concentrations in the air, water, and soil samples were 2.12 × 10^−2^ pg/m^3^ (range 4.77 × 10^−3^–4.04 × 10^−2^ pg/m^3^), 6.66 × 10^−2^ ng/L (range 3.32 × 10^−2^–0.119 ng/L), and 1.96 × 10^−3^ ng/g dry weight (dw) (range 6.76 × 10^−4^–7.44 × 10^−3^ ng/g dw), respectively. In a previous study, the mean NBFRs concentrations (the sums of the PBT, HBB, and PBEB concentrations) in air samples from Weifang and Nanning in China were 4.2 × 10^3^ and 11.9 pg/m^3^, respectively [27]. In Arctic water samples, the mean concentrations of PBBz and PBT were 1.5 and 0.02 ng/L, respectively [28]. HBB, PBEB, and PBT concentrations of 8672, 132, and 20.6 ng/g wet weight (ww), respectively, have been found in sediment from e-waste dismantling sites in southern China [29]. Sun et al. found a mean concentration of 16 NBFRs of 389 ng/g in dust from Hangzhou, China [30]. HBB, PBT, and PBEB concentrations of 2.1, 0.04, and 0.02 ng/g dw, respectively, have been found in soil from an e-waste dismantling site in Australia [31]. The NBFRs concentrations in the abiotic environmental samples of Xilingol grassland were low. There are no known NBFRs production facilities or e-waste dismantling sites in the Xilingol area. Therefore, the NBFRs found in Xilingol grassland samples may be transported to the study area through long-distance transport. The NBFRs concentrations in the biota samples are shown in Figure 2. The mean NBFRs concentration in the four plant samples was 0.0209 ng/g dw (range 5.04 × 10^−3^–4.09 × 10^−^^2^ ng/g dw). The NBFRs concentrations in the plant samples decreased in the order *Allium mongolicum* (0.0286 ng/g dw) > *Leymus chinensis* (0.0225 ng/g dw) > *Artemisia frigida* (0.0231 ng/g dw) > *Eleusine indica* (0.0136 ng/g dw). In a previous study, pTBX, PBT, PBBz, and HBB concentrations of 0.7–5.8, 0.4–8.3, 0.4–9.4, and 0.2–7.0 ng/g, respectively, were found in willow bark samples from the upper reaches of the Yellow River in China [32].

Insects are an important part of the terrestrial food chain. The mean NBFRs concentration in the four insect samples was 0.0437 ng/g lipid weight (lw) (range 0.0266–0.0785 ng/g lw). The NBFRs concentrations in the insect samples decreased in the following order: moth (0.0502 ng/g lw) > locust (0.0486 ng/g lw) > scarab (0.0392 ng/g lw) > carabidae (0.0382 ng/g lw). The NBFRs concentrations in the non-insect animal tissue samples decreased in the following order: cuckoo muscle (mean 1.11 ng/g lw) > weasel muscle (mean 0.901 ng/g lw) > snake muscle (mean 0.277 ng/g lw) > sparrow muscle (0.248 ng/g lw) > lizard (mean 0.209 ng/g lw) > sheep muscle (mean 0.160 ng/g lw) > swallow muscle (mean 0.150 ng/g lw) > toad (0.131 ng/g lw) > mouse muscle (mean 0.0496 ng/g lw). HBB, PBT, and PBEB concentrations of 3099, 106, and 4.14 ng/g lw, respectively, have been found in freshwater food web samples from near e-waste dismantling sites in southern China [29]. NBFRs concentrations in fish consumed by people in France showed that the PBEB, PBT, HBB, PBBz, and pTBX concentrations were <limit of detection (LOD)-0.166, <LOD-19.82, <LOD-38.06, <LOD-6.39, and <LOD-1.90 pg/g ww, respectively [33]. The NBFRs concentrations in the animal samples from the Xilingol grassland were lower than concentrations previously found in animal samples from polluted areas but were higher than the concentrations found in edible fish in France, indicating that NBFRs pollution in remote areas and NBFRs enrichment by animals cannot be ignored. NBFRs concentrations in animal and human hair samples were also determined. The mean NBFRs concentrations in the cow hair, horse hair, and wool samples were 1.05 × 10^−^^2^, 5.23 × 10^−3^, and 7.44 × 10^−3^ ng/g dw, respectively. The mean NBFRs concentration in the human hair samples was 8.63 × 10^−^^2^ ng/g dw, which was higher than the animal hair.

### 3.2. NBFRs Congener Patterns in the Different Xilingol Grassland Samples

**Figure 3:**
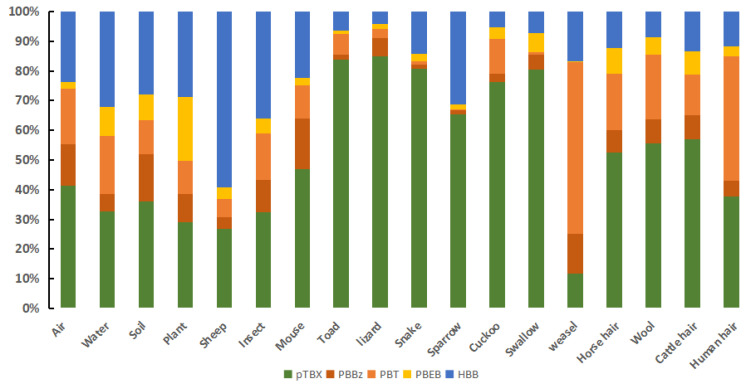
Novel brominated flame retardant contributions to the total novel brominated flame retardant concentrations in the samples from grasslands in Inner Mongolia, China.

The contributions of the individual NBFRs to the total NBFRs concentrations in the samples from the Xilingol grassland are shown in Figure 3. The dominant NBFRs in the air (pTBX 41.3%, HBB 23.7%), water (pTBX 32.6%, HBB 32.2%), soil (pTBX 36.0%, HBB 27.9%), and plant (pTBX 29.1%, HBB 28.7%) were pTBX and HBB. The dominant NBFRs in the insect samples were HBB and pTBX, which contributed 36.0% and 32.2% of the total NBFRs concentrations, respectively. The pTBX contributed 83.7%, 84.8%, 80.7%, and 46.8% of the total NBFRs concentrations in the toad, lizard, snake, and mouse samples, respectively. pTBX was the dominant NBFRs in the bird samples. pTBX contributed 65.5%, 76.2%, and 80.3% of the total NBFRs concentrations in the sparrow, cuckoo, and swallow samples, respectively. pTBX was also the dominant NBFRs in the horse hair, wool, and cow hair samples, contributing 52.5%, 55.6%, and 56.8%, respectively, of the total NBFRs concentrations. However, HBB contribution (59.1%) was higher for the sheep samples than the other animal samples. PBT and HBB were the dominant NBFRs in the weasel samples, contributing 49.8% and 14.4%, respectively, of the total NBFRs concentrations. PBT and pTBX were the dominant NBFRs in the human hair samples, contributing 41.8% and 37.6%, respectively, of the total NBFRs concentrations. These results indicated that pTBX contributed markedly more to the total NBFRs concentration in amphibians and reptiles (lizard, snake, and toad) and birds than in the abiotic samples and that PBT or HBB contributed markedly more to the total NBFRs concentration in the mammals (sheep, weasel and human hair) than in the abiotic samples. This indicated that NBFRs are selectively bioaccumulated to different degrees by different animals.

**Figure 4:**
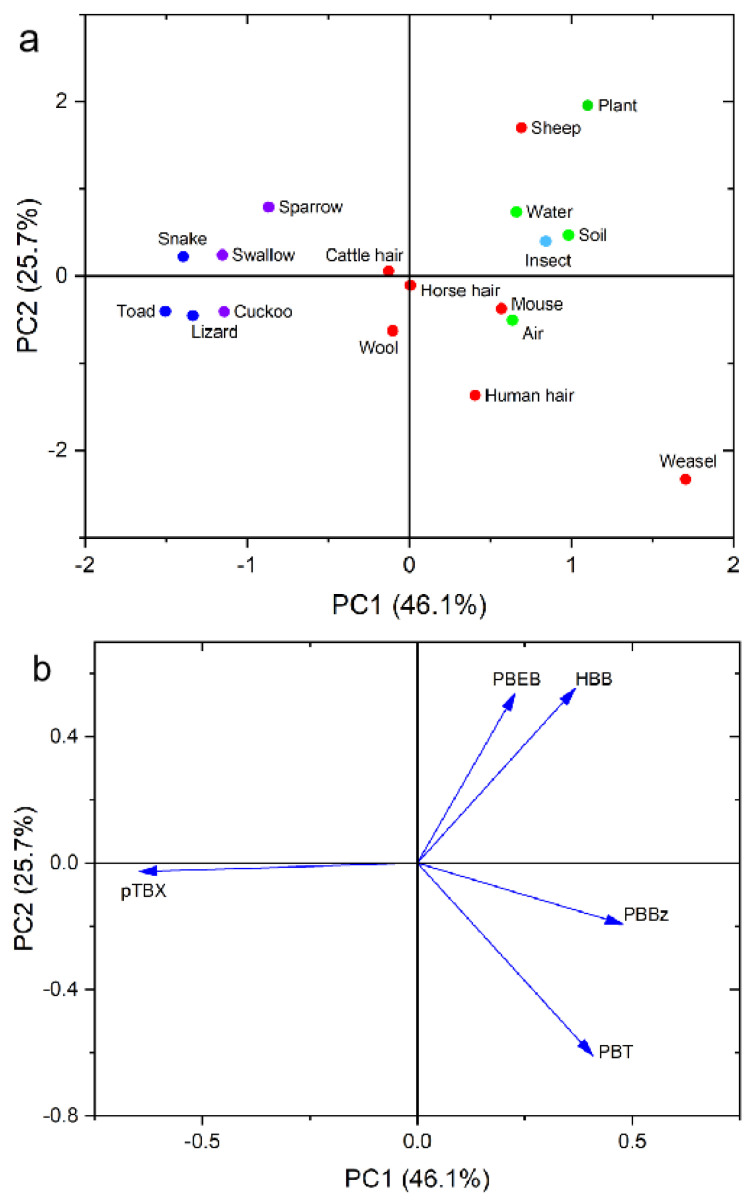
Two−dimensional principal component analysis: (**a**) score plot and (**b**) factor loading plot for the novel brominated flame retardant concentrations in the samples from grasslands in Inner Mongolia, China.

Principal component analysis (PCA) was performed to identify similarities and differences between the NBFRs patterns in the different samples. The individual NBFRs concentrations were converted into percentage contributions to the total NBFRs concentrations before PCA was performed. Principal components (PCs) with eigenvalues >1 were used. Two PCs explained 71.8% of the variance, as shown in Figure 4a. The PCA factor loading plot is shown in Figure 4b. PBBz and PBT had high positive PC1 loads (0.479 and 0.408, respectively), and pTBX had a high negative PC1 load (−0.646), meaning that high PC1 values were mainly related to PBBz and PBT, and low PC1 values were mainly related to pTBX. PC1 accounted for 46.1% of the variance, and PC2 accounted for 25.7% of the variance. HBB had a high positive PC2 load (0.511) and PBT had a high negative PC2 load (−0.609), meaning that high PC2 values were mainly related to HBB and low PC2 values were mainly related to PBT. The datapoints for the amphibians and reptiles (lizard, snake, and toad) and birds (cuckoo, sparrow, and swallow) were on the left of the PCA score map, the datapoints for the other mammal samples (cow hair, horse hair, human hair, mouse, sheep, weasel, and wool) were on the lower right of the map, and the datapoints for the abiotic environmental media samples (air, soil, and water), grass samples, and insect samples were on the upper right of the map. This indicated that the NBFRs were bioaccumulated to different extents in the amphibians, reptiles and birds, and in the other mammals. This may have been related to the different diet habits and metabolic modes of the different animals.

### 3.3. NBFRs Biomagnification in the Grassland Food Chain in Inner Mongolia

Biomagnification factors (BMFs) are often used to evaluate the biomagnification of chemicals in predators from their prey [34]. BMFs are suitable for assessing terrestrial ecosystems [35]. BMFs are defined as the ratio between the concentration of a pollutant in the predator and the concentration of the pollutant in the food consumed by the predator. A BMF > 1 indicates that biomagnification has occurred [36]. The BMFs of NBFRs for the Xilingol grassland food chain are shown in Table 1. In the insect-grass food chain, the BMFs of NBFRs were less than 1, indicating that no biomagnification occurred. In sheep (muscle)-grass food chain, HBB had the highest biomagnification (BMFs = 5.57). In the weasel (muscle)-mouse food chain, PBT had the highest biomagnification (BMFs = 27.9), followed by HBB (BMFs = 4.23). In the human hair-wool/cow hair/horse hair food chain, each individual NBFR had high biomagnification (BMFs > 5). However, in the food chain where birds (sparrow, swallow and cuckoo) were predators, pTBX had the greatest biomagnification (BMFs > 5). Similarly, pTBX had the greatest biomagnification in the food chain where amphibians and reptiles were predators (BMFs > 2). The results imply that NBFRs congeners show different biomagnification in different food chains, which may be due to the biological bioaccumulation and metabolic characteristics of animal species, or the different physical and chemical properties of NBFRs.

In the current limited research on biomagnification of NBFRs, the BMFs values of NBFRs in Lake Ontario trout were PBT (5.63), PBEB (2.03), PBBz (0.94), and pTBX (0.46), respectively [37]. The BMFs values of HBB and PBT in snake/frog were less than 1 [38]. The BMFs of NBFRs showed significant differences between aquatic organisms and terrestrial organisms. The physiological differences between aquatic animals and terrestrial animals may affect the metabolism of NBFRs in different species, and then affect its biomagnification.

### 3.4. NBFRs Bioaccumulation in the Organs and Tissues of Sheep

**Figure 5:**
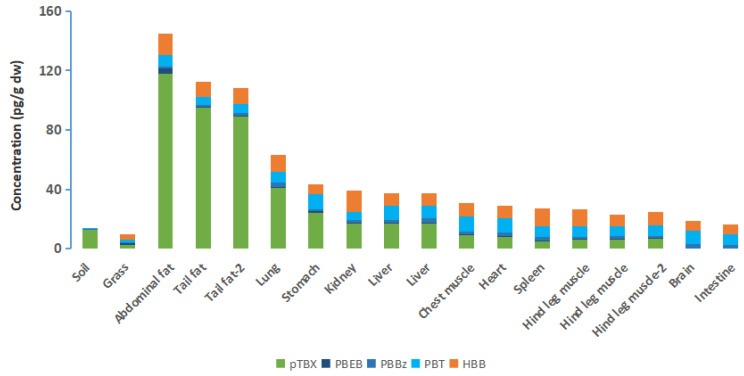
Novel brominated flame retardant concentrations in the different organs and tissues of sheep from Inner Mongolia grassland, China.

NBFRs (pTBX, PBEB, PBBz, PBT, and HBB) were detected in organs and tissues of grassland sheep. The sum of pTBX, PBEB, PBBz, PBT, and HBB concentrations in the organs and tissues of sheep were 16.3–145 pg/g dw (61.3–956 pg/g lw). For each component of NBFRs, the mean concentrations of pTBX, HBB, PBT, PBBz, and PBEB in sheep organs and tissues were 28.8 (<LOQ-118) pg/g dw, 9.72 (6.20–14.6) pg/g dw, 7.74 (5.38–10.3) pg/g dw, 1.95 (1.19–2.69) pg/g dw, and 0.717 (0.285–3.30) pg/g dw, respectively. In the organs and tissues of the sheep, adipose tissue (abdominal fat 145 pg/g dw, tail fat 113 pg/g dw) contained the highest concentration of NBFRs, followed by lung (63.4 pg/g dw). NBFRs concentration in the organs and tissues decreased in the following order: stomach (43.5 pg/g dw) > kidney (39.3 pg/g dw) > liver (37.2 pg/g dw) > chest muscle (31.0 pg/g dw) > heart (29.2 pg/g dw) > spleen (27.2 pg/g dw) > hind leg muscle (24.6 pg/g dw) > brain (18.9 pg/g dw) > small intestine (16.3 pg/g dw) (Figure 5).

In the above study, it was found that NBFRs had biomagnification in sheep (muscle)-grass food chain, but NBFRs did not show biomagnification in wool. This shows that the biomagnification of NBFRs in different parts of the sheep body is different, so it is necessary to further study the biomagnification of NBFRs in various organs and tissues of sheep.

The BMFs values of NBFRs in various organs and tissues of grassland sheep were calculated (Table 2); the BMFs value is equal to the concentrations of NBFRs in tissues or organs divided by the concentrations of grass. The BMFs values of pTBX were, in descending order: intake organs (lung 17.3 and stomach 10.3) > metabolic and excretory organs (liver 7.22 and kidney 7.22) > other organs (heart 3.41, spleen 2.24, brain 0 and small intestine 0). The pTBX had high biomagnification in directly exposed intake organs. However, BMFs decreased significantly in other non-external exposed organs (heart, spleen, brain and small intestine). It shows that pTBX is more easily accumulated in sheep fat. However, pTBX was not detected in sheep brain, showing the different accumulation of pTBX in different tissues and organs of sheep. The BMFs value of PBT was greater than 2 in intake organs (lung 3.52 and stomach 5.05), metabolic and excretory organs (liver 4.63 and kidney 2.79), and other organs (heart 4.56, spleen 3.77, brain 4.48 and small intestine 3.57). The BMFs values of HBB were also greater than 2 for intake organs (lung 3.73 and stomach 2.12), metabolic and excretory organs (liver 2.54 and kidney 4.52), and other organs (heart 2.75, spleen 3.67, brain 2.16. The BMFs values of PBBz were greater than 1 for intake organs (lung 2.98 and stomach 1.49), metabolic and excretory organs (liver 2.40 and kidney 1.99), and other organs (heart 3.15, spleen 2.61, brain 3.03 and small intestine 2.80). The BMFs values of PBT, HBB, and PBBz had little difference in sheep organs. This may indicate that sheep organs can accumulate PBT, HBB, and PBBz. In particular, they have high accumulation in sheep brain, which is worthy of our attention in regard to whether they will bring corresponding health risks. The BMFs values of PBEB in all organs and tissues of sheep (except abdominal fat) was less than 1. This indicates that PBEB does not have biomagnification in sheep organs. Therefore, when studying biomagnification, it is difficult to draw a true conclusion only by studying a single tissue or organ; in particular, the biomagnification of adipose tissue may not be able to determine whether chemicals have biomagnification in organisms.

## 4. Conclusions

NBFRs (pTBX, HBB, PBT, PBBz, and PBEB) were detected in various environmental media from the Xilingol grassland, indicating that NBFRs can be transported over long distances to this remote area. The pTBX was more likely to be bioaccumulated and biomagnified in the food chain of amphibians, reptiles, and birds, whereas PBT and HBB were more likely to be bioaccumulated and biomagnified in the food chain of mammals. This may be because these animals have different eating habits and metabolic patterns. The distribution of NBFRs in sheep visceral organs is affected by organ function and physical and chemical properties of NBFRs. Sheep adipose tissue can continuously bioaccumulate NBFRs. According to the concentration distribution of NBFRs in sheep organs and tissues, PBT, HBB, and PBBz easily bioaccumulated in sheep. The biomagnification effect of sheep adipose tissue and internal organs on NBFRs was inconsistent, so the biomagnification of chemicals in organisms cannot be determined only by the biomagnification of adipose tissue.

To our knowledge, this is the first measurement of NBFRs in the grassland food chain and sheep organs. Our preliminary results show that NBFRs remain ubiquitous, and animal species and tissue types can affect the biomagnification of NBFRs. By studying the distribution of NBFRs in different types of grassland animals and sheep tissues, we can further improve our understanding of the bioaccumulation of NBFRs in organisms and investigate the potential ecological risks of these non-banned chemicals. However, due to the limitation of the number of samples, the bioconcentration of NBFRs in the grassland food chain still needs more research.

## Figures and Tables

**Table 1:** Novel brominated flame retardant biomagnification factors for the samples from grassland in Inner Mongolia.

Type	Consumer (Part)	Food (Part)	pTBX	PBBz	PBT	PBEB	HBB
Insect	Insect (whole)	Grass	0.654	0.640	0.823	0.132	0.719
Mammals	Mouse (muscle)	Grass	1.05	1.09	0.551	0.0963	0.436
	Sheep (muscle)	Grass	2.49	1.14	1.47	0.500	5.57
	weasel (muscle)	Mouse (muscle)	1.31	4.08	27.9	0.663	4.23
	Wool	Grass	0.680	0.310	0.693	0.0951	0.109
	Cattle hair	Grass	0.978	0.436	0.609	0.188	0.233
	Horse hair	Grass	0.452	0.200	0.426	0.0991	0.108
	Human hair	Wool	7.85	7.55	22.3	6.89	15.6
	Human hair	Cattle hair	5.46	5.37	25.4	3.49	7.32
	Human hair	Horse hair	11.8	11.7	36.4	6.60	15.7
Birds	Sparrow (muscle)	Grass	5.98	0.349	0.0611	0.211	2.91
	Swallow (muscle)	Insect (whole)	9.54	1.94	0.192	5.12	0.793
	Cuckoo (muscle)	Insect (whole)	34.3	3.93	11.0	11.1	2.27
Amphibians and reptiles	Toad (whole)	Insect (whole)	7.61	0.467	0.357	0.756	0.530
	lizard (whole)	Insect (whole)	5.23	1.41	0.408	0.734	0.501
	Snake (muscle)	Mouse (muscle)	2.55	0.221	0.157	1.14	1.07
	Snake (muscle)	Sparrow (muscle)	0.450	0.694	1.41	0.519	0.161
	Snake (muscle)	Swallow (muscle)	0.432	0.195	0.546	0.162	0.820
	Snake (muscle)	Cuckoo (muscle)	0.120	0.0963	0.00951	0.0749	0.286
	Snake (muscle)	Toad (whole)	0.541	0.811	0.294	1.10	1.23
	Snake (muscle)	Lizard (whole)	0.787	0.268	0.257	1.13	1.30

**Table 2:** NBFRs biomagnification factors in the organs and tissues of sheep.

Tissues and Organs	pTBX	PBT	PBBz	HBB	PBEB
Abdominal fat	49.8	3.71	1.86	4.61	2.67
Tail fat	40.2	2.64	1.39	3.23	0.562
Tail fat-2	37.6	3.07	1.42	3.42	0.597
Lung	17.3	3.52	2.98	3.73	0.669
Stomach	10.3	5.05	1.49	2.12	0.676
Kidney	7.22	2.79	1.99	4.52	0.362
Liver	7.22	4.63	2.40	2.54	0.433
Liver	7.14	4.21	3.25	2.62	0.532
Chest muscle	3.98	4.78	2.31	2.97	0.410
Heart	3.41	4.56	3.15	2.75	0.290
Hind leg muscle	2.59	3.31	1.85	3.59	0.382
Hind leg muscle	2.70	3.35	2.01	2.35	0.382
Hind leg muscle-2	2.78	3.35	2.03	2.82	0.456
Spleen	2.24	3.77	2.61	3.67	0.231
Brain	0.00	4.48	3.03	2.16	0.302
Intestine	0.00	3.57	2.80	1.95	0.324

## Data Availability

Data is contained within the article.
